# Towards an integrative model of C_4_ photosynthetic subtypes: insights from comparative transcriptome analysis of NAD-ME, NADP-ME, and PEP-CK C_4_ species

**DOI:** 10.1093/jxb/eru100

**Published:** 2014-03-18

**Authors:** Andrea Bräutigam, Simon Schliesky, Canan Külahoglu, Colin P. Osborne, Andreas P.M. Weber

**Affiliations:** ^1^Institute of Plant Biochemistry, Cluster of Excellence on Plant Sciences (CEPLAS), Heinrich-Heine-University, Universitätsstrasse 1, D-40225 Düsseldorf, Germany; ^2^Department of Animal and Plant Sciences, University of Sheffield, Sheffield S10 2TN, UK

**Keywords:** C4 photosynthesis, *Dichanthelium clandestinum*, *Megathyrsus maximus*, PEP-CK, RNA-Seq, transcriptomics.

## Abstract

C_4_ photosynthesis affords higher photosynthetic carbon conversion efficiency than C_3_ photosynthesis and it therefore represents an attractive target for engineering efforts aiming to improve crop productivity. To this end, blueprints are required that reflect C_4_ metabolism as closely as possible. Such blueprints have been derived from comparative transcriptome analyses of C_3_ species with related C_4_ species belonging to the NAD-malic enzyme (NAD-ME) and NADP-ME subgroups of C_4_ photosynthesis. However, a comparison between C_3_ and the phospho*enol*pyruvate carboxykinase (PEP-CK) subtype of C_4_ photosynthesis is still missing. An integrative analysis of all three C_4_ subtypes has also not been possible to date, since no comparison has been available for closely related C_3_ and PEP-CK C_4_ species. To generate the data, the guinea grass *Megathyrsus maximus*, which represents a PEP-CK species, was analysed in comparison with a closely related C_3_ sister species, *Dichanthelium clandestinum*, and with publicly available sets of RNA-Seq data from C_4_ species belonging to the NAD-ME and NADP-ME subgroups. The data indicate that the core C_4_ cycle of the PEP-CK grass *M. maximus* is quite similar to that of NAD-ME species with only a few exceptions, such as the subcellular location of transfer acid production and the degree and pattern of up-regulation of genes encoding C_4_ enzymes. One additional mitochondrial transporter protein was associated with the core cycle. The broad comparison identified sucrose and starch synthesis, as well as the prevention of leakage of C_4_ cycle intermediates to other metabolic pathways, as critical components of C_4_ metabolism. Estimation of intercellular transport fluxes indicated that flux between cells is increased by at least two orders of magnitude in C_4_ species compared with C_3_ species. In contrast to NAD-ME and NADP-ME species, the transcription of photosynthetic electron transfer proteins was unchanged in PEP-CK. In summary, the PEP-CK blueprint of *M. maximus* appears to be simpler than those of NAD-ME and NADP-ME plants.

## Summary

An integrative analysis of all three C_4_ subtypes is presented. It is shown that PEP-carboxykinase represents an addition to the NAD- or NADPH-malic enzyme subtypes but not a distinct subtype.

## Introduction

Plants using C_4_ photosynthesis display higher carbon conversion efficiency than C_3_ plants (Amthor, 2010) and are thus among the most productive crop plants. C_4_ plants also dominate many natural ecosystems because this trait enables efficient growth under water- and nitrogen-limited conditions at high temperatures. As the area of available arable land decreases and the human population increases, C_4_ photosynthesis has become a trait of high potential for a second green revolution (Hibberd *et al.*, 2008; Maurino and Weber, 2013). To recreate this complex trait efficiently by synthetic approaches, a mechanistic understanding of the genetic architecture controlling the biochemical, anatomical, and regulatory aspects of C_4_ photosynthesis is required. Although the enzymes of the core cycle were discovered >50 years ago, knowledge about the metabolism underlying the C_4_ trait remains incomplete. The engineering potential of C_4_ metabolism was explored in the guinea grass *Megathyrsus maximus*.

C_4_ photosynthesis increases photosynthetic efficiency by concentrating CO_2_ at the site of Rubisco using a biochemical carbon-concentrating mechanism that is distributed between two compartments, the mesophyll cell (MC)and the bundle sheath cell (BSC), in most known C_4_ species. The trait has convergently evolved at least 60 times (Sage *et al.*, 2011) and always employs phospho*enol*pyruvate carboxylase (PEPC) to incorporate bicarbonate into phospho*enol*pyruvate (PEP), yielding the four-carbon molecule oxaloacetate (OAA). For transfer to the site of Rubisco, OAA is converted to either malate by reduction or aspartate by transamination. Different evolutionary lineages, however, have different means to decarboxylate the now-organic carbon to release the CO_2_ at the site of Rubisco: NADP-dependent malic enzyme (ME) decarboxylates malate to pyruvate in chloroplasts; NAD-ME decarboxylates malate to pyruvate in mitochondria; and phospho*enol*pyruvate carboxykinase (PEP-CK) decarboxylates OAA to PEP in the cytosol. The resulting C_3_ acid is then transported back to the site of PEPC as PEP in the case of PEP-CK-based decarboxylation, or as pyruvate or alanine for NAD-ME and NADP-ME. In the chloroplasts, pyruvate is recycled to PEP by the action of pyruvate, phosphate dikinase (PPDK) with the reaction products pyrophosphate and AMP recycled by pyrophosphorylase (PPase) and AMP kinase (AMK). Historically, three different metabolic C_4_ types were proposed based on the decarboxylation enzyme: the NADP-ME type, the NAD-ME type, and the PEP-CK type, of which the latter was considered the most complex (Hatch, 1987). An NADP-ME C_4_-type leaf and an NAD-ME C_4_-type leaf have been compared with closely related C_3_ species globally at the transcriptome level which identified core C_4_ cycle components and placed upper limits on the number of genes changed transcriptionally in C_4_ metabolism (Bräutigam *et al.*, 2011; Gowik *et al.*, 2011).

Among the C_4_ plants with the highest contribution of PEP-CK activity to decarboxylation is the guinea grass *M. maximus*, one of the plant species in which the enzyme activity was originally described and therefore a prototypical PEP-CK plant (summarized in Hatch, 1987). *Megathyrsus maximus* has been taxonomically regrouped several times (Grass Phylogeny Working Group II, 2012), and has also been called *Panicum maximum* and *Urochloa maxima*. Other species with high PEP-CK activity in addition to NAD-ME activity are *Urochloa panicoides* (Ku *et al.*, 1980) and *Chloris gayana* (Hatch, 1987).

The biochemical characterization of PEP-CK-type C_4_ plants identified carboxylation by PEPC as in all other C_4_ plants (Ku *et al.*, 1980) and two decarboxylation enzymes, PEP-CK and NAD-ME (Ku *et al.*, 1980; Chapman and Hatch, 1983; Burnell and Hatch, 1988a, *b*; Agostino *et al.*, 1996). Exclusive decarboxylation by PEP-CK has not been reported to date. Carboxylation and decarboxylation are linked by the transfer acids malate, aspartate, alanine, pyruvate, and PEP (summarized in Hatch, 1987). In *C. gayana*, the distribution of transfer acids has been investigated by feeding labelled CO_2_; both malate and aspartate became rapidly labelled, indicating that both are used as transfer acids. Furthermore, the labelling rate of aspartate was twice as high as that of malate, indicating an approximate flux ratio of 2:1 between aspartate and malate (Hatch, 1979). In *M. maximus*, the aminotransferase enzyme activities were localized to the cytosol (Chapman and Hatch, 1983) and the malate-producing malate dehydrogenases (MDHs) were present as both chloroplastidic NADP-MDH and cytosolic and mitochondrial NAD-MDH (Chapman and Hatch, 1983).

A high rate of PEP-CK decarboxylation is linked to malate decarboxylation in the bundle sheath and consumption of the resulting reducing equivalents (REs) either by reduction of OAA to malate or by the mitochondrial electron transport chain (Hatch, 1987; Burnell and Hatch, 1988a, *b*). The ATP produced is exported to the cytosol to fuel the PEP-CK reaction (Hatch *et al.*, 1988). It remains unresolved whether pyruvate kinase activity produces pyruvate from PEP (Chapman and Hatch, 1983) for transfer back to the mesophyll.

PEP-CK enzyme activity has also been reported for several NADP-ME and NAD-ME species: (Walker *et al.*, 1997; Wingler *et al.*, 1999; Bräutigam *et al.*, 2011; Pick *et al.*, 2011; Sommer *et al.*, 2012; Christin *et al.*, 2013; Muhaidat and McKown, 2013). Whether PEP-CK is an independent subtype or whether it is essentially similar to NAD-ME or NADP-ME species remains unresolved. Supplemental PEP-CK activity was apparently favoured during the evolution of C_4_ plants, possibly because it lowers the concentrations and gradients of the transfer acids (Wang *et al.*, 2014), but it is unknown whether it is beneficial for engineering the trait.

*Megathyrsus maximus* displays a classical Kranz anatomy with large BSCs and few MCs between bundles (Yoshimura *et al.*, 2004). In this arrangement, the cell types are linked by plasmodesmata, which allow symplastic transport of the transfer acids along the concentration gradient (Evert *et al.*, 1977; Hatch, 1987; Botha, 1992; Bräutigam and Weber, 2011). However, this dependence upon symplastic transport has been questioned (Sowinski *et al.*, 2008) and the gradients measured between the cell types in maize do not quite reach the required steepness (Stitt and Heldt, 1985). In *M. maximus*, the photosynthetic rate is correlated with growth light intensity and with plasmodesmatal density (Sowinski *et al.*, 2007). The large BSCs have increased organelle number compared with C_3_ BSCs and their chloroplasts have fully developed grana (Yoshimura *et al.*, 2004). As a consequence of linear electron transfer in the bundle sheath chloroplasts, oxygen is produced, leading to higher photorespiration compared with other C_4_ plants (Furbank and Badger, 1982; Ohnishi and Kanai, 1983; Farineau *et al.*, 1984). However, the quantum yield for *M. maximus* is comparable with, or above, the quantum yield for *Zea mays* (NADP-ME+PEP-CK) and *Sorghum bicolor* (NADP-ME) (Ehleringer and Pearcy, 1983). Neither the intercellular transport rates of transfer acids nor the global consequences of linear electron transfer in BSCs have been explored.

The recent sequencing of the model plant *Setaria italica* (Bennetzen *et al.*, 2012) and the detailed phylogenetic analysis of grasses (Grass Phylogeny Working Group II, 2012) enables RNA-Seq of the PEP-CK subtype of C_4_ photosynthesis, by providing a mapping reference and the identification of suitable sister species, respectively. Although the phylogeny of the Paniceae tribe of grasses is not resolved with complete confidence (Grass Phylogeny Working Group II, 2012), the C_3_ grass *Dichanthelium clandestinum* and the PEP-CK C_4_ grass *M. maximus* are currently considered as monophyletic lineages that shared the last common ancestor 18±4 Myr (million years) ago (Vicentini *et al.*, 2008; Grass Phylogeny Working Group II, 2012). *Dichanthelium clandestinum* is therefore among the closest living sister taxa to the PEP-CK-type model species *M. maximus* and was chosen for the comparison in the work reported here.

Two complementary strategies were chosen to extend the blueprint of C_4_ photosynthesis to associated pathways and functions beyond the core cycle, which has already been described for the NAD-ME plant *C. gynandra* (Bräutigam *et al.*, 2011): (i) a broad analysis of C_4_-related functions using comparative RNA-Seq data for PEP-CK (Paniceae, this study), NADP-ME (*Flaveria* species) (Gowik *et al.*, 2011 *a*), and NAD-ME (*Cleome* species) (Bräutigam *et al.*, 2011), and leaf RNA-Seq data sets for *Z. mays* (Li *et al.*, 2011), *S. italica* (Bennetzen *et al.*, 2012), *S. bicolor*, *Oryza sativa*, and *Brachypodium distachyon* (Davidson *et al.*, 2012); and (ii) a detailed C_3_ versus C_4_ comparison between the PEP-CK species *M. maximus* and its C_3_ sister species *D. clandestinum.*

## Materials and methods

### Plant growth and harvesting

*Megathyrsus maximus* (Collection of the Botanical Garden Düsseldorf) and *D. clandestinum* (grown from seed obtained from B&T World Seeds, Perpignan, France) plants were grown with 16h of light at 24 °C. *Dichanthelium clandestinum* was maintained vegetatively. Harvesting was scheduled to the eight-leaf stage, which was 3–5 weeks after germination or tiller initiation. In the middle of the light period, the third leaf from the top—the third youngest—was sampled in three replicates for sequencing (one for 454 and two for Illumina sequencing) and five replicates for enzyme activities, and quenched in liquid nitrogen immediately after cutting. Pools of 20 plants per sample were harvested.

### Enzyme activities

C_4_ decarboxylation enzymes were extracted from frozen, ground leaves using 1ml of buffer [25mM TRIS-HCl (pH 7.5), 1mM MgSO_4_, 1mM EDTA, 5mM dithiothreitol (DTT), 0.2mM phenylmethylsulphonyl fluoride (PMSF), and 10% (v/v) glycerol] per 10mg of leaf powder. After desalting using NAP-5 size exclusion columns, enzyme activities of PEP-CK (Walker *et al.*, 1995), NAD-ME, and NADP-ME (Hatch and Mau, 1977) were measured photometrically based on the absorption change of NAD(P)H at 340nm.

### CO_2_ assimilation rates and isotope discrimination

For three replicates of both species, the net leaf photosynthetic assimilation rate (*A*) was measured using a Li-Cor LI-6400XT infrared gas exchange analyser (LI-COR Inc., Lincoln, NE, USA). CO_2_-dependent assimilation curves (*A*–*C*_i_) were measured at 1500 μmol m^–2^ s^–1^ constant light. Light-dependent assimilation curves were measured at a constant external CO_2_ concentration of 400 ppm.

For ^13^C isotope discrimination, leaf powder was dried and analysed using the isotope ratio mass spectrometer IsoPrime 100 (IsoPrime Ltd, Cheadle, Manchester, UK). Results were expressed as relative values compared with the international standard (Vienna-PeeDee Belemnite).

### RNA extraction and sequencing

Isolation of total RNA from ground tissue of *M. maximus* was performed using a guanidium thiocyanate extraction followed by an ethanol and a lithium chloride precipitation, as described by Chomczynski and Sacchi (1987). Extraction of total RNA from *D. clandestinum* was performed using a TRIS-borate buffer to cope with large amounts of polysaccharides, as described by Westhoff and Herrmann (1988). mRNA for 454 library preparation was enriched by using Qiagen Oligotex poly(A)-binding silicone beads and further prepared for sequencing as described in Weber *et al.* (2007). For Illumina sequencing two replicates of total RNA were used per sample. Library preparation and sequencing were carried out according to the manufacturer’s suggestions by the local NGS facility (BMFZ, Biologisch-Medizinisches Forschungszentrum, Düsseldorf), using Roche Titanium chemicals for 454 and the TruSeq library kit for Illumina HiSeq 2000. Long and short read raw data were submitted to the short read archive (SUB440021, *D. clandestinum*; SUB439950, *M. maximus*).

### Sequence assembly and expression statistics

*De novo* assembly was done using CAP3 (Huang and Madan, 1999) using default parameters on cleaned 454 reads. Reads were cleaned by trimming low quality ends, discarding reads of overall minor quality, and removal of exact duplicates using scripts of the FASTX-Toolkit (http://hannonlab.cshl.edu/fastx_toolkit/) as described in Schliesky *et al.* (2012). Contigs were annotated by BLAST best hit mapping to *S. italica* (v164) representative coding sequences. Quantitative expression was determined by mapping of all Illumina reads against *S. italica* representative coding sequences (v164) using BLAT (Kent, 2002) and counting the best hit for each read. Zero counts were treated as true 0. Expression was normalized to reads per mappable million and per kilobase (rpkm) *Setaria* CDS. Eight rpkm were chosen as the threshold of expression to discriminate background transcription. Differential expression was determined by DESeq (Anders and Huber, 2010), a negative binomial test, in R (R Development Core Team, 2012). A significance threshold of 0.05 was applied after Bonferroni correction for multiple hypothesis testing and is reported in Supplementary Table S3 available at *JXB* online. For all single genes mentioned in the text, changes in expression were confirmed using the 454 data set which was also mapped across species to *S. italica* as described in Bräutigam *et al*. (2011) (Supplementary Table S3). Pathway enrichment was determined by Benjamini–Hochberg correction (Benjamini and Hochberg, 1995). Fisher’s exact test was used to test for over-/under-representation of MapMan categories.

### Meta comparison of functional categories

Expression data for *B. dystachyon*, *S. bicolor*, and *O. sativa* were previously published by Davidson *et al.* (2012). Transcript sequences for mature *Z. mays* leaves (+4cm sample) were obtained from the short read archive SRA012297 (Li *et al.*, 2010) and mapped to *S. italica* representative coding sequences. Expression data for five Flaveria species were taken from Gowik *et al.* (2011). Expression data for *Cleome gynandra* (C_4_) and *Tarenaya hassleriana* (C_3_) were taken from Bräutigam *et al*. (2011). The samples were produced in different laboratories and with different sequencing technologies. Only the presence of C_4_-related traits was interpreted, as absence calls may be due to inconsistent sampling with regard to leaf developmental state, time of day, and other variables.

EC (enzyme classifiers; Schomburg *et al.*, 2013) and Pfam (protein family; Sonnhammer *et al.*, 1997) annotations were added to the two reference transcriptomes, *S. italica* CDS (v164) and *Arabidopsis thaliana* CDS (TAIR10). Reduction of data complexity to functional classifiers was achieved by summing up all expression values mapping to the same EC or Pfam. Venn diagram sets were built through logical operators; that is, expression is higher/lower in all C_4_ versus C_3_ comparisons (see also Supplementary Table S2 at *JXB* online). Comparison pairs were chosen according to the sequencing method and experimenter: *M. maximus* versus *D. clandestinum* (this study), *S. bicolor* versus *O. sativa* and versus *B. dystachyon* (all from Davidson *et al.* 2012); *Z. mays* (Li *et al.* 2011) and *S. italica* (Bennetzen *et al.* 2012) were orphan data sets as no comparison partner was sequenced with the same technology and both were compared against *B. dystachion* as the C_3_ reference. The dicots were compared as previously published (Bräutigam *et al.* 2011; Gowik *et al.*, 2011).

### Leaf cross-sections for confocal microscopy

Fresh mature leaves (upper third of the leaf) of *M. maximus* and *D. clandestinum* were cut transversally and fixed in PBST [1× PBS buffer (137mM NaCl, 2.7mM KCl, 4.3mM Na_2_HPO_4_, 1.4mM KH_2_PO_4_); 1% (v/v) Tween-20; 3% (v/v) glutaraldehyde] overnight at room temperature. Leaf cross-sections were stained with 0.1% 4’,6-diamidino-2-phenylindole (DAPI) solution in phosphate-buffered saline (PBS) for 30min. Subsequently, cross-sections were analysed with an LSM 780 (Zeiss) confocal microscope with a ×40 objective. Z-stack images were processed with LSM Zeiss software to produce maximum intensity overlay images.

## Results

### *D. clandestinum* is well suited for a C_3_ comparison with *M. maximus*

**Fig. 1.**
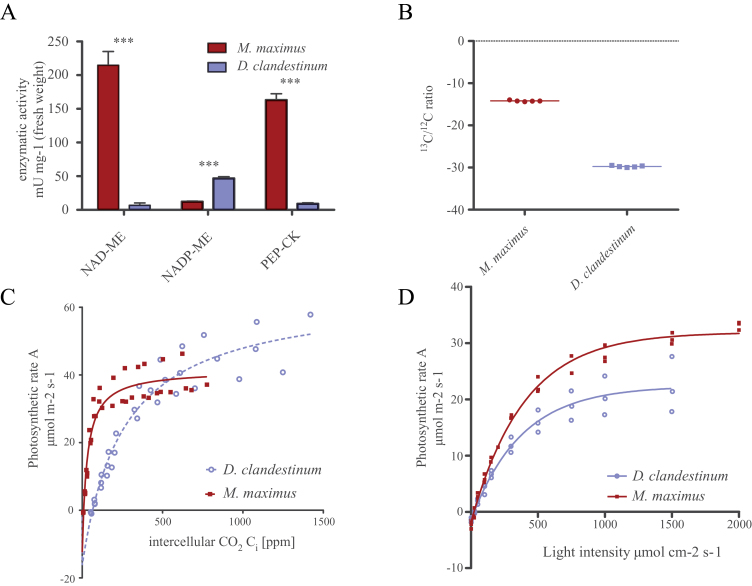
Physiological characterization of *Megathyrsus maximus* and *Dicanthelium clandestinum*. Activity of the decarboxylation enzymes in *M maximus* and *D. clandestinum* (A); ^13^C/^12^C stable isotope ratio (B); *A*–*C*_i_ curves at 1500 μE (C); and light curves at 400 ppm CO_2_ (D). ***P<0.001. (This figure is available in colour at *JXB* online.)

The PACMAD clade of the grasses is exceptionally rich in C_4_ plants (Christin *et al.*, 2013) to the point that it is difficult to identify and cultivate closely related C_3_ species for comparative analyses. To confirm that *D. clandestinum* is a *bona fide* C_3_ plant and to confirm the biochemical subtype of the C_4_ plant *M. maximus*, different parameters were tested. The measured enzyme activities, stable isotopic carbon discrimination, *A*–*C*_i_ curves, and light curves indicated that *D. clandestinum* indeed represents a C_3_ plant (Fig. 1). *Megathyrsus maximus* has high NAD-ME and PEP-CK enzyme activities as compared with *D. clandestinum*, but comparable activities of the NADP-ME decarboxylation enzyme (Fig. 1A). *Dichanthelium clandestinum* discriminates against ^13^C at a δ^13^C ratio of –30‰, while *M. maximus* shows C_4_ typical relaxation of carbon isotope discrimination with a δ^13^C ratio of –13‰ (Fig. 1B). The *A*–C_i_ curve of *M. maximus* shows a low CO_2_ compensation point of 9 ppm and saturation of the net carbon fixation rate at 41 μmol m^–2^ s^–1^. The *A*–*C*_i_ curve of *D. clandestinum* plants grown alongside *M. maximus* indicates a CO_2_ compensation point of 65 ppm and does not saturate even with high CO_2_ concentrations, as is typical for a C_3_ plant (Fig. 1C). The light response curves of CO_2_ assimilation show similar rates for both types of plants at very low light intensities, with *M. maximus* continuously outgaining *D. clandestinum* as light increases. Thus, *M. maximus* has slightly higher quantum efficiency and saturates at a higher light intensity compared with *D. clandestinum* (Fig. 1D). In summary, the physiological data indicate that *D. clandestinum* is a suitable comparison partner for *M. maximus* due to its phylogenetic proximity and physiological characteristics typical of C_3_ plants.

### Quantitative and qualitative transcriptome information

The transcriptomes of both grass species were determined by RNA-Seq using two complementary technologies to gain quantitative gene expression information and provide a sequence resource optimized for C_4_ unigene assembly. RNA-Seq libraries from two biological replicates of *M. maximus* and two biological replicates of *D. clandestinum* were sequenced with Illumina HiSeq2000 technology and yielded upwards of 53 million reads per replicate, of which >48 million reads were of high quality (Table 1). Reads were mapped cross-species to a closely related reference sequence database derived from the *S. italica* genome (Bennetzen *et al.*, 2012) and between 66% and 74% of reads matched the reference sequence database (Table 1). In the reference sequence database, 13 043 genes were matched with >8 rpkm, of which 792 were detected as differentially up-regulated in C_4_ and 376 were detected as differentially down-regulated in C_4_ (Table 1). In addition, 1.1 million and 0.9 million 454/Roche Titanium reads were generated and assembled for *M. maximus* and *D. clandestinum*, respectively, and mapped onto *S. italica* as a quality control for the Illumina mapping. The majority of gene expression differences followed similar trends in the 454 mapping or were not detected among the 454 reads; only 12 genes displayed inversely regulated patterns with the different sequencing technologies. Reads were filtered and trimmed based on a Phred score of 30 and assembled with CAP3 (Huang and Madan, 1999) to provide a reliable database of unigenes. C_4_ cycle genes were covered by unigenes with full length (Supplementary Table S1 at *JXB* online). About 40 000 unigenes were generated for each species (Table 1).

**Table 1.** *Sequencing, mapping, and assembly statistics for* Megathyrus maximus *and* Dicanthelium clandestinum

Read mapping	*Megathyrus maximus* 1	*Megathyrus maximus* 2	*Dicanthelium clandestinum* 1	*Dicanthelium clandestinum* 2
No. of Illumina reads	61 703 536	56 780 148	53 079 709	56 765 538
No. of cleaned reads	56 470 008	52 282 627	48 160 148	50 328 269
Mappable reads (%)	41 570 126 (73.6%)	38 848 638 (74.3%)	34 151 633 (70.9%)	33 311 704 (66.2%)
No. of 454 reads	1 152 766		971 065	
No. of contigs in assembly	39 565		40 320	
*Setaria* CDS with >8 rpkm	13 043			
Differentially up-regulated	792			
Differentially down-regulated	376			

### Genes commonly up- or down-regulated in all C_4_ decarboxylation types

Comparative RNA-Seq data for NADP-ME species versus C_3_ sister species (Gowik *et al.*, 2011) and for NAD-ME species versus C_3_ sister species (Bräutigam *et al.*, 2011), three RNA-Seq data sets for *S. bicolor*, *O. sativa*, and *B. dystachyon* from one comparative experiment (Davidson *et al.*, 2012), as well as orphan RNA-Seq data sets for two PACMAD NADP-ME grasses, *Z. mays* (Li *et al.*, 2011) and *S. italica* (Li *et al.*, 2011), are publicly available. By combining the public data with data from this study, the up- and down-regulated core C_4_ genes altered in all C_4_ species were identified.

**Fig. 2.**
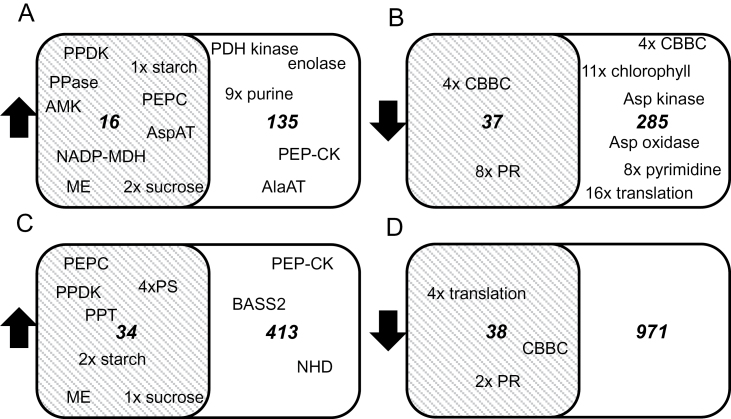
Shared expression based on function in NAD-ME (white set) versus all C_4_ species (grey set). Up- and down-regulated functions are based on expression of functions represented by enzyme classifiers (EC) (A, B) and by Pfam domain combinations (PDC) (C, D). PPDK, pyruvate phosphate dikinase; PPase, inorganic pyrosphate phosphorylase; AMK, adenosine monophosphate kinase; PEPC, phospho*enol*pyruvate carboxylase; AspAT, aspartate aminotransferase; MDH, malate dehydrogenase; ME, malic enzyme; PDH, pyruvate dehydrogenase; PEP-CK, phospho*enol*pyruvate carboxykinase; AlaAT, alanine aminotransferase; CBBC, Calvin–Benson–Bassham cycle; PR, photorespiration; Asp, aspartate; PPT, phospho*enol*pyruvate phosphate translocator; PS, photosynthesis; BASS2, pyruvate transporter; NHD sodium proton antiporter; all functions are listed in Supplementary Table S2 at *JXB* online.

Gene by gene comparisons may be limited between different C_3_–C_4_ species comparison pairs since for known C_4_ genes, most notably PEPC, recruitment of paralogous genes has already been demonstrated (Westhoff and Gowik, 2004; Besnard *et al.*, 2009; Christin and Besnard, 2009). In addition, a function may be distributed among multiple genes, each of which singly does not appear changed. To overcome the inherent limitations of orthologous gene pair comparisons when analysing multiple species pairs, reads were summed to categories which represent a function rather than a particular gene. Enzymes were identified in the reference species *A. thaliana* and *S. italica* on the basis of EC numbers which cover ~5000 different enzymes (Schomburg *et al.*, 2013), of which 1073 are present in the references, and reads for each gene were summed based on the EC number. For example, reads mapping to different isogenes encoding PEPC are no longer represented by the gene identifier but they have been collapsed onto the EC number representing PEPC function (4.1.1.31). All proteins in both reference species were also assigned to their protein family on the basis of Pfam domains (Sonnhammer *et al.*, 1997), of which 4073 unique combinations are present in the references, and reads for each gene were summed based on the Pfam domain combination. Consequently, PEPC is no longer represented by a gene identifier but its function is represented by its Pfam domain combination pf00311. The functions up-regulated or down-regulated in all C_4_ species compared with their related C_3_ species and those limited to the two NAD-ME type based species were then analysed (Fig. 2A–D; Supplementary Table S2 at *JXB* online).

The functional analysis based on EC numbers indicated a consistent up-regulation of 16 functions in all C_4_ comparisons. The C_4_ enzymes with PPDK, PPase, AMK, PEPC, aspartate aminotransferase (AspAT), NADP-dependent malate dehydrogenase (NADP-MDH), and ME are up-regulated in all comparisons (Fig. 2A). In addition, one function related to starch synthesis, two functions related to sucrose synthesis, and six functions currently unlinked to C_4_ were identified (Fig. 2A; Supplementary Table S2 at *JXB* online). Both NAD-ME species have 135 up-regulated functions in common, including PEP-CK, alanine aminotransferase (AlaAT), pyruvate dehydrogenase (PDH) kinase, and nine enzymes involved in purine synthesis and turnover (Fig. 2A). The 37 functions down-regulated in all C_4_ comparisons include four of the Calvin–Benson (CBB) cycle and eight related to photorespiration (Fig. 2B). The down-regulated functions in both NAD-ME-type comparisons included aspartate kinase and aspartate oxidase, eight functions of pyrimidine synthesis, four of the CBB cycle, 11 of chlorophyll synthesis, and 16 of translation (Fig. 2B).

The functional analysis based on Pfam domain combinations showed 34 up-regulated functions in all C_4_ species including PEPC, PPDK, phospho*enol*pyruvate phosphate translocator (PPT), and ME. Four photosystem-related functions, two functions related to starch synthesis, and one related to sucrose synthesis are also among those up-regulated (Fig. 2C). The 413 NAD-ME-type related up-regulated functions include PEP-CK, the pyruvate transporter (BASS2, Furumoto *et al.*, 2011), and the sodium:hydrogen antiporter (NHD; Furumoto *et al.*, 2011), all detected with high fold changes (Fig. 2C; Supplementary Table S2 at *JXB* online). Among the 38 down-regulated functions are the CBB cycle, photorespiration, and translation (Fig. 2D).

The analyses of C_4_-related functions extend the known C_4_ up-regulated traits to sucrose and starch synthesis and the C_4_ down-regulated traits to the CBB cycle, photorespiratory functions, and translation. They also provide candidates for as yet unknown functions which may be C_4_ related. The NAD-ME-type related functions include those that prevent the leakage of C_4_ cycle metabolites into general metabolism.

### The PEP-CK decarboxylation subtype is qualitatively similar to but quantitatively distinct from the NAD-ME

Given the blueprint of NAD-ME C_4_ photosynthesis (Bräutigam *et al.*, 2011), it was tested whether the differentially regulated functions in the PEP-CK species are those already identified for the NAD-ME species. The occurrence of PEP-CK activity in species previously classified as NADP-ME and NAD-ME species and recent modelling efforts raised the question of whether the classification of PEP-CK as its own C_4_ type is warranted (Wang *et al.*, 2014).

The C_4_ genes were extracted from the complete data set (Supplementary Table S3 at *JXB* online) and compared with those of *C. gynandra* (Bräutigam *et al.*, 2011). *Megathyrsus maximus* and *C. gynandra* show elevated expression of enzymes and transporters known to be required for C_4_ photosynthesis (Table 2). *Megathyrsus maximus* showed significantly increased transcripts encoding BASS2, NHD, PPDK, and PPT, which is similar to the dicotyledonous NAD-ME C_4_ species *C. gynandra*. In comparison with a C_3_ reference, the up-regulation of these transcripts was between 27-fold and 67.5-fold in *M. maximus* and between 15-fold and 226-fold in *C. gynandra*. PPDK induction, however, was lower in *M. maximus* compared with *C. gynandra*, which might indicate increased regeneration of PEP by PEP-CK rather than PPDK. Both species also showed changes in AMK and PPase expression, but these were not expressed to a significantly higher extent in *M. maximus*. The NHD and AMK expressed at high levels are paralogous to the same proteins required for the C_4_ cycle in the dicotyledonous plant (Table 2). The carboxylation enzyme PEPC was significantly up-regulated in both the dicot and the monocot, again using paralogues (Table 2). For the generation of the C_4_ transfer acids malate and aspartate, only cytosolic AspAT was significantly up-regulated in *M. maximus*, while no up-regulation of the cytosolic isozyme was observed in *C. gynandra*. Cytosolic targeting was determined by localization prediction of the full-length protein of *M. maximus* (Supplementary Table S4 at *JXB* online). The most abundant transcript encoding MDH also encoded a cytosolic isozyme, suggesting use of the NAD-MDH form (Supplementary Table S4).

**Table 2.** *The expression of C*_*4*_ *cycle genes of* Megathyrsus maximus *in comparison with* Dicanthelium clandestinum *and* Cleome gynandra*, and their location in* M. maximus

Module	Gene name	*Setaria* ID	Function	Predicted location of translated protein	*M. maximus* expression (rpkm)	*D. clandestinum* expression (rpkm)	Fold- change	Significantly changed (DESeq, Bonferroni)	Fold change of function in *C. gynandra*
Regeneration	BASS2	Si001591m	Pyruvate sodium symport	Chloroplast	2797	69	40.5	Yes	87.3
	NHD	Si029362m	Sodium proton antiport	Chloroplast	838	31	27.0	Yes	**15.9**
	PPDK	Si021174m	Pyruvate→PEP	Chloroplast	13380	283	47.3	Yes	226.4
	PPa	Si017993m	Pyrophosphate→phosphate	Chloroplast	450.5	158.5	2.8	NS	3.2
	AMK	Si017707m	AMP→ADP	Chloroplast	985.5	114.5	8.6	NS	**8.9**
	PPT	Si013874m	PEP phosphate antiport	Chloroplast	405	6	67.5	Yes	15.0
Carboxylation	PEPC	Si005789m	PEP→OAA	Cytosol	18393	303.5	60.6	Yes	**77.6**
C_4_ transfer acid	AspAT	Si001361m	Asp↔OAA	Cytosol	1273	79	16.1	Yes	2^*a*^
	GAP-DH	Si014034m	3-GPA→TP	Cytosol	4544	1538	3.0	NS	0.2
	MDH	Si036550m	Malate↔OAA	Cytosol	735	452	1.6	NS	0.44^*a*^
Decarboxylation NAD-ME	DIC	Si014081m	Malate phosphate antiport	mitochondrion	455	114	4.0	NS	519.0
	PIC	Si017569m	Phosphate proton symport	Mitochondrion	225	96	2.3	NS	2.5
	ME	Si000645m& Si034747m^*b*^	Malate→pyruvate	Mitochondrion	1299	230	5.6	NS	20.3
	Unknown/diffusion?		Pyruvate export						
Decarboxylation PEP-CK	PEP-CK	Si034404m	OAA→PEP	Cytosol	8819	99	89.5	Yes	8.6
	AAC	Si017474m	ATP ADP/P antiport	Mitochondrion	461	150	3.1	NS	0.4

Bold indicates use of a paralogous gene.

NS, non significant.

^*a*^ A paralogue in a different compartment is up-regulated.

^*b*^ Reads map to both malic enzymes

Two different decarboxylation modules using NAD-ME and PEP-CK, respectively, are active in the plants (Fig. 1A). In *M. maximus*, neither the transport protein DIC, responsible for antiport of malate into mitochondria against phosphate (Palmieri *et al.*, 2008), and PIC, responsible for symport of phosphate and protons (Pratt *et al.*, 1991; Hamel *et al.*, 2004), nor the decarboxylation enzyme NAD-ME were significantly changed, although all were up-regulated between 2.3- and 5.6-fold (Table 2). This is in stark contrast to the up-regulation detected for DIC and NAD-ME in *C. gynandra* which was between 20-and 519-fold. No candidate for pyruvate export from the mitochondria could be identified. The situation is reversed for the PEP-CK module where PEP-CK was significantly up-regulated 90-fold in *M. maximus* but only 8.6-fold in *C. gynandra*. The mitochondrial ATP-ADP translocase, AAC (Haferkamp *et al.*, 2002), is up-regulated in *M. maximus*, but not to a significant degree (Table 2). Orthologous AlaATs are significantly up-regulated by 37-fold in both species. Unlike the *C. gynandra* protein, which is predicted to be targeted to mitochondria, the *M. maximus* protein is predicted to be cytosolic (Supplementary Table S4 at *JXB* online). The *M. maximus* AlaAT protein showed a shortened N-terminus when aligned to the *S. italica* gene (Supplementary Table S4), hence *in silico* targeting predicted cytosolic localization. Finally, non-significant up-regulation of TPT and plastidic GAP-DH was detected in *M. maximus* to comparable levels as in *C. gynandra* (Table 2).

In addition to single gene analysis, differentially regulated genes were subjected to pathway enrichment analysis to detect changes in gene expression for whole pathways such as the CBB cycle, photorespiration, and photosynthesis. None of the pathways was significantly enriched among the differentially regulated genes (Supplementary Table S5 at *JXB* online).

The gene-by-gene and enrichment analyses revealed a similar but not identical blueprint for the PEP-CK species compared with the NAD-ME species. The core cycle blueprint was amended to include a companion transporter for the malate phosphate antiporter DIC, which couples it to the proton gradient with phosphate proton symport through PIC.

### Energy requirements derived from the PEP-CK blueprint

**Fig. 3.**
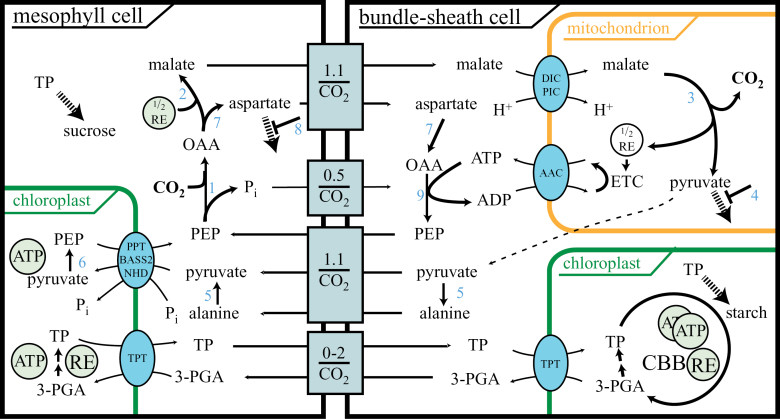
Extended model for NAD-ME with high PEP-CK activity. Transport modules, consisting of one or more transporters, are shown together with the net transport through the module. Abbreviations: (1) Phospho*enol*pyruvate carboxylase; (2) malate dehydrogenase; (3) NAD-dependent malic enzyme (NAD-ME); (4) pyruvate dehydrogenase kinase; (5) alanine aminotransferase; (6) pyruvate, phosphate dikinase; (7) aspartate aminotransferase; (8) aspartate oxidase and aspartate kinase; (9) phospho*enol*pyruvate carboxykinase (PEP-CK); 3-PGA, 3-phosphoglyceric acid; TP, triose-phosphate; CBB, Calvin–Benson–Bassham cycle; OAA, oxaloacetic acid; RE, reducing equivalent; BASS2, pyruvate transporter; NHD, sodium proton antiporter; PPT, phospho*enol*pyruvate phosphate translocator; TPT, triose-phosphate phosphate translocator; ETC, electron transfer chain. Dashed arrows represent leakage to general metabolism. (This figure is available in colour at *JXB* online.)

The energy requirements of intracellular transport reactions were not considered when the energy balance of C_4_ photosynthesis was originally calculated (i.e. Kanai and Edwards, 1999), although pyruvate transport was hypothesized to be active based on measurements of the metabolite concentration gradients in maize (Stitt and Heldt, 1985). To assess the energy requirements of the PEP-CK-based C_4_ cycle, the amended blueprint was translated into a model of PEP-CK C_4_ photosynthesis (Fig. 3).

Energy requirements are calculated following one turn of the cycle (Fig. 3): after PEP is carboxylated to OAA, half of the OAA is reduced to malate (Hatch *et al.*, 1988), requiring on average 0.5 REs derived from photosynthesis for each CO_2_ (Fig. 3). The remaining OAA is transported as aspartate (Fig. 3). At the bundle sheath mitochondria, malate exchange for phosphate via DIC is coupled to the proton gradient via phosphate proton symport by PIC (Fig. 3). This process consumes the proton gradient of mitochondria. The proton gradient is also used to drive mitochondrial ATP synthesis for the PEP-CK reaction which decarboxylates OAA to PEP (Fig. 3) and is regenerated by oxidizing the NADH produced by malate decarboxylation (Fig. 3). The carboxylation, transfer, and decarboxylation thus consume on average 0.5 NADPH per CO_2_ generated in photosynthetic electron transfer. During regeneration, the PPDK reactions require 2 ATP for the regeneration of each pyruvate but, since only half of the flux runs through malate decarboxylation and therefore pyruvate, only 1 ATP is required for each CO_2_. The PPDK reaction is driven towards PEP regeneration by the PPase, which splits the energy-rich bond of pyrophosphate and makes the PPDK reaction irreversible *in vivo*. The production of PEP and its export through PPT creates the proton gradient required to import pyruvate and cycle sodium through the transport system (Fig. 3). Although the active transport of pyruvate is driven by the proton gradient, it requires no additional input of energy beyond that expended for the PPDK reaction (Furumoto *et al.*, 2011). The regeneration phase thus requires 1 ATP in total. The CBB cycle requires 3 ATP and 2 REs from photosynthesis, which may be consumed in the bundle sheath or mesophyll.

The total PEP-CK-based C_4_ cycle, assuming no overcycling, thus requires 4 ATP and 2.5 NADPH from the photosynthetic electron transfer chain while solely NADP-ME-based C_4_ photosynthesis requires 5 ATP and 2 NADPH and C_3_ photosynthesis requires 3 ATP and 2 NADPH for each CO_2_ (Kanai and Edwards, 1999). Engineering a PEP-CK-type C_4_ cycle will thus avoid the adjustments required for the photosynthetic electron transfer chain since the demands in terms of the ATP and NADPH ratio are almost the same as in C_3_ plants.

### Intercellular transport derived from the PEP-CK blueprint

Engineering a C_4_ cycle may require modifications to the symplastic transport interface (Weber and Bräutigam, 2013). To estimate the difference in intercellular transport for each MC, intercellular transport events between C_4_ and C_3_ were compared. Data from the scheme depicted in Fig. 3 were combined with anatomical data (Supplementary Fig. S1 at *JXB* online) and photosynthetic rates (Fig. 1C).

Since transport events are assessed per MC and not per leaf area, the number of MCs per leaf area was determined. In the C_4_ plants, photosynthesis requires the MC and its adjacent BSC; in the C_3_ plant, each MC is a self-contained unit. Microscopic imaging of leaf cross-sections revealed typical Kranz anatomy in *M. maximus* with large BSCs, each of which was connected to multiple MCs (Supplementary Fig. S1 at *JXB* online). The density of MCs was almost twice as high in the C_3_ leaf compared with the C_4_ leaf (Table 3). Since the photosynthetic rate per leaf area is also higher in *M. maximus* (Fig. 1C), almost twice as much CO_2_ is fixed in each MC–BSC pair in *M. maximus* compared with an MC of *D. clandestinum* (5.4 versus 2.6 pmol CO_2_ per unit and second). In *D. clandestinum*, only sucrose transport is required across the MC wall. Since each sucrose molecule carries 12 carbons, and since only half of the carbon is exported at any given time, with the remainder stored as starch, the assimilation of one molecule of CO_2_ requires 1/12×1/2=0.042 transport events in the C_3_ plant (Table 3). In contrast, the PEP-CK-based C_4_ cycle requires between 2.75 and 4.75 transport events depending on the extent of RE shuttling because the C_4_ acids, the C_3_ acids, balancing phosphates, and REs are transported (Table 3). The total number of transport events is estimated by multiplying the number of CO_2_ molecules assimilated with the number of transport events required for each CO_2_ as 11.6–20.1 pmol s^–1^ in the C_4_ species while for C_3_ it is 0.1 pmol s^–1^. C_4_ photosynthesis requires between 100- and 200-fold more transport events than C_3_ photosynthesis, such that the intercellular transport capacity needs to be increased by approximately two orders of magnitude in C_4_ (Table 3).

**Table 3.** *Parameters for the calculation of transport requirements for the PEP-CK/NAD-ME C*_*4*_ *cycle show that C4 photosynthesis requires 100–200 times more transport events*Cell density was estimated from Supplementary Fig. S1 at *JXB* online and divided by photosynthetic parameters derived from Fig. 1 to yield the photosynthetic rate per cell (A). C_4_ cycle transport requirements were derived from Fig. 3 and summed to calculate total transport events (B). Total transport events through plasmodesmata are calculated as A×B.

		*M. maximus*	*D. clandestinum*
Photosynthetic parameter	Photosynthetic cell density (Giga photosynthetic units m^–2^)	6.987	12.5
	Photosynthetic rate at 400 ppm (μmol m^–2^ s^–1^)	29.6	20.8
A	Photosynthetic rate CO_2_ per cell (pmol CO_2_ pu^–1^ s^–1^)	4.2	1.7
Metabolic parameter (transport events per CO_2_)	C_4_ acid (malate, aspartate)	1.1	
	C_3_ acid (PEP, pyruvate, alanine)	1.1	
	Phosphate balance (P_i_; 50% PEP assumed)	0.55	
	RE shuttle (triose-phosphate, 3-PGA)	0–2	
	Sucrose export		0.042
B	Total no. of transport events (transport events CO_2_^–1^ pu^–1^)	2.75–4.75	0.042
A×B	No. of transport events per cell (pmol transport events s^–1^)	11.6–20.0	0.1

Engineering of the C_4_ cycle will thus almost certainly require engineering of the BSC–MC interface, as it is highly unlikely that an existing C_3_ MC could support the >100-fold increased symplastic flux.

## Discussion

### Assembly and mapping characteristics

This study was designed to compare two closely related C_3_ and C_4_ species to increase the probability of detecting C_4_-related rather than species-related differences. While for several C_3_ grass species, such as rice and *Brachypodium*, the genomes have already been sequenced and thus could serve as C_3_ reference for comparative transcriptome sequencing, all of these belong to the BEP clade and have thus diverged 45–55 Myr ago from *M. maximus* (Grass Phylogeny Working Group II, 2012), which belongs to the PACMAD clade. *Dichanthelium clandestinum* was chosen as a C_3_ species from within the PACMAD clade for the transcriptomic comparison presented here. Although the precise phylogenetic position of the *Dichanthelium* clade of Paniceae, which includes *D. clandestinum*, has not been determined, it was recently placed as sister to the group, which contains *M. maximus* (Grass Phylogeny Working Group II, 2012), with a divergence time of 14–22 Myr ago (Vicentini *et al.*, 2008). For quantification of steady-state transcript amounts, the RNA-Seq reads were mapped onto the coding sequences predicted from the *Setaria* genome. The closer phylogenetic proximity of *M. maximus* to *Setaria* is represented in the slightly higher mapping efficiency of its reads (Table 1). Overall, the mapping efficiency is above that of the *Flaveria* species on *Arabidopsis* (Gowik *et al.*, 2011 *a*) but below that of the *Cleomaceae* on *Arabidopsis* (Bräutigam *et al.*, 2011). The disadvantage of a slightly uneven mapping efficiency was, however, outweighed by mapping reads from both species onto a common genome-based reference sequence, which enabled normalization to reads per kilobase per million reads. In addition, low abundance transcripts are frequently under-represented in contig assemblies, while high abundance transcripts were fragmented into multiple contigs per transcript. Establishing orthology, while possible with tools such as OrthoMCL, requires assumptions about similarities. Mapping onto a reference database as previously successfully established (Bräutigam *et al.*, 2011, Gowik *et al.*, 2011) was chosen to overcome this problem.

Contig assembly from Illumina reads results in fragmented contigs, especially for the high abundance contigs, as observed previously in other RNA-Seq projects (Bräutigam and Gowik, 2010; Franssen *et al.*, 2011; Schliesky *et al.*, 2012). The C_4_ transcripts are among the most highly expressed transcripts in leaves of C_4_ plants (Bräutigam *et al.*, 2011). To produce high confidence contigs, the transcriptome was sequenced by a long read technology, the reads cleaned with a high base quality threshold of Phred=30, and assembled with CAP3. Within the database, full-length contigs for all candidate C_4_ genes were identified (Supplementary Tables S1, S4 at *JXB* online), validating a hybrid approach to quantification and database generation (Bräutigam and Gowik, 2010).

### Are NAD-ME and the PEP-CK distinct subtypes of C_4_ photosynthesis?

The three classical subtypes of C_4_ photosynthesis, NADP-ME, NAD-ME, and PEP-CK, have been analysed by comparative transcriptome sequencing (Bräutigam *et al.*, 2011; Gowik *et al.*, 2011; this study). If the two C_4_ types NAD-ME and PEP-CK which both rely wholly or partially on NAD-ME-based decarboxylation were fundamentally different, major differences in the transcriptional profile would be expected. However, quantification of transcript abundance showed that the functions up-regulated in the NAD-ME plant *C. gynandra*, which shows some PEP-CK activity (Sommer *et al.*, 2012), and the PEP-CK plant *M. maximus*, which displays high PEP-CK activity, are quite similar.

The bicarbonate acceptor regeneration module is essentially identical. Both plant species belong to the sodium pyruvate transport group, as defined by Aoki *et al.* (1992), and show joint up-regulation of not only the sodium pyruvate symporter BASS2 (Furumoto *et al.*, 2011), but also the companion sodium:hydrogen antiporter NHD, and the PEP phosphate antiporter PPT (Bräutigam *et al.*, 2011; Gowik *et al.*, 2011; Table 2). The generation of the transfer acids appears to be cytosolic as neither of the two plastidial dicarboxylate transporters, DiT1 (OAA/malate antiporter) (Weber *et al.*, 1995; Kinoshita *et al.*, 2011) and DiT2 (OAA/aspartate antiporter) (Renne *et al.*, 2003), was up-regulated (Supplementary Table S3 at *JXB* online) and the most abundant contigs encoding AspAT and MDH were predicted to be cytosolic (Table 2; Supplementary Table S4). The cytosolic localization relaxes the need to up-regulate organellar transporters, which are required to import substrates and export products. The two species use differentially localized AspATs, a mitochondrial isozyme in the case of *C. gynandra* (Sommer *et al.*, 2012) and a cytosolic one in the case of *M. maximus* (Table 2; Toledo-Silva *et al.*, 2013). For the decarboxylation process, both species use a combination of PEP-CK and NAD-ME and consequently have the same functions up-regulated. The degree of up-regulation, however, mirrors the enzyme activity differences, with PEP-CK transcripts being much more induced in *M. maximus* and NAD-ME and associated transporters much more induced in *C. gynandra* (Table 2). Hence the difference in decarboxylation biochemistry between both species rests in an altered balance between NAD-ME and PEP-CK activities, while the overall pathway is very similar.

At least part of the C_3_ acid transport is accomplished through alanine to balance the amino groups between MCs and BSCs. The up-regulated AlaAT for both plants is an orthologous pair, which is targeted to organelles in *C. gynandra* (Bräutigam *et al.*, 2011; Sommer *et al.*, 2012) and *S. italica* (Supplementary Table S4 at *JXB* online). However, enzyme activity measurements placed high AlaAT activity in the cytosol of *M. maximus* (Chapman and Hatch, 1983). The *in silico* translation of the *M. maximus* transcript revealed that it encodes a truncated version of AlaAT in comparison with the *Setaria* gene, in which a potential start ATG in-frame with the coding sequence is prefaced by a stop codon. The shortened protein is predicted to be cytosolic (Supplementary Table S4). Hence, the cytosolic AlaAT activity in *M. maximus* appears to have evolved by loss of the target peptide of an originally organellar-targeted protein. The simpler cycle suggests that the *M. maximus* blueprint is easier to engineer compared with the blueprints of NAD-ME (Bräutigam *et al.*, 2011; Sommer *et al.*, 2012) and NADP-ME species (Gowik *et al.*, 2011; Pick *et al.*, 2011; Denton *et al.*, 2013; Weber and Bräutigam, 2013).

Multiple species which had previously been grouped as NADP-ME or NAD-ME plants have different degrees of PEP-CK activity (Walker *et al.*, 1997; Pick *et al.*, 2011; Sommer *et al.*, 2012; Muhaidat and McKown, 2013) and modelling shows the advantages of supplemental PEP-CK activity in conferring environmental robustness to the pathway (Wang *et al.*, 2014), raising the question as to whether PEP-CK-type plants deserve their own group. While the functions up-regulated in *C. gynandra* and *M. maximus* are similar, there are differences with regard to localization of the enzymes generating the transfer acids. Whether the different enzyme localizations are tightly associated with the type and degree of use of the decarboxylation enzymes remains to be determined once additional transcriptomes are sequenced and a global view is enabled on more than just one prototypical species for each historical C_4_ type. For engineering, it is probably advisable to follow the blueprint of a particular species since it is currently not clear whether differences in transfer acid generation are only species specific or are tied to other processes such as decarboxylation enzymes and therefore functionally relevant.

### An extended model of C_4_ photosynthesis with high PEP-CK activity

Understanding the evolution of C_4_ metabolism and re-engineering a C_4_ cycle in a C_3_ plant requires a mechanistic understanding of the parts making up the system (Denton *et al.*, 2013). The global transcriptomics analysis of *M. maximus* compared with *D. clandestinum* enabled the extension of the C_4_ metabolism model presented earlier for *M. maximus* (Hatch, 1987) and *C. gynandra* (Bräutigam *et al.*, 2011; Sommer *et al.*, 2012).

#### Transport processes and core cycle

The *M. maximus* analysis confirmed DIC as the mitochondrial malate importer (Table 2; Bräutigam *et al.*, 2011). The companion transporter, which couples malate transport to the proton gradient of the mitochondria and supplies mitochondria with inorganic phosphate for ATP production, is probably PIC (Hamel *et al.*, 2004; Table 2; Fig. 3). The only transporter which remains unknown at the molecular level is the mitochondrial pyruvate exporter. The candidate pyruvate transport protein, the human mitochondrial pyruvate carrier (MPC) (Bricker *et al.*, 2012; Herzig *et al.*, 2012), is not differentially expressed in *C. gynandra* and *M. maximus*. Potentially, pyruvate can traverse biomembranes in its protonated form by simple diffusion (Benning, 1986), although this is unlikely in a cellular context given that only one out of 10^5^ molecules of pyruvic acid occurs in the protonated form at physiological pH values. Although early models did not take a reducing equivalent shuttle across both chloroplast envelopes into account for PEP-CK species (Hatch, 1987), possibly because *M. maximus* lacks chloroplast dimorphism (Yoshimura *et al.*, 2004), measurements of enzyme activity confirmed glyceraldehyde dehydrogenase in both MCs and BSCs of *U. panicoides* (Ku and Edwards, 1975), and RNA-Seq indicated modest up-regulation of the necessary transporters and enzymes (Table 2). Engineering a C_4_ cycle will critically depend on correctly enabling the transport of substrates through transporters and companion transporters (Weber and von Caemmerer, 2010; Fig. 3). Balancing reducing power between MCs and BSCs via triose-phosphate/phosphate translocators in chloroplasts in both MCs and BSCs appears also to be required in species which lack chloroplast dimorphism (Table 2; Yoshimura *et al.*, 2004).

Knowledge about the intracellular transport proteins involved in C_4_ photosynthesis has recently improved significantly (compare with Weber and von Caemmerer, 2010; Bräutigam and Weber, 2011; Denton *et al.*, 2013; Weber and Bräutigam, 2013), largely due to RNA-Seq-enabled identification and characterization of the chloroplast pyruvate transporter (Furumoto *et al.*, 2011), and the placement of several known transport proteins in the C_4_ pathway (Taniguchi *et al.*, 2003; Bräutigam *et al.*, 2011; Gowik *et al.*, 2011 *a*; Kinoshita *et al.*, 2011). However, information about the intercellular transport has not progressed since the discovery of sieve element-like plasmodesmata plates in the MC–BSC interface (Evert *et al.*, 1977; Botha, 1992).

The difference in total transport events between the C_3_ and the C_4_ species was estimated using the data provided by the model shown in Fig. 3, by images of the cellular architecture (Supplementary Fig. S1 at *JXB* online), and by photosynthetic rate measurements (Fig. 1C). The large difference in the requirement for intracellular transport between C_4_ and C_3_ pathways is not predominantly driven by the rather small differences in photosynthetic rates (Fig. 1C), but by two other factors: the number of MCs per leaf area and the number of transport events required for each CO_2_ assimilated. The large BSCs, each of which borders several MCs, and the fact that *M. maximus* requires two cells in each photosynthetic unit means that the C_3_ grass has almost twice as many photosynthetic units in the same leaf area. The net CO_2_ assimilation capacity is thus not only higher by the ~20% higher photosynthetic rate per leaf area but—if normalized to the number of MCs—is almost twice as high for each unit. The second factor is the number of transport processes occurring over each interface. Intercellular transport for each C_3_ cell is very low, 0.042 events per CO_2_ assimilated for an MC. The transport events for the C_4_ cycle are more difficult to estimate since, in addition to the comparatively fixed flux of C_4_ and C_3_ acids in the cycle, the PEP-balancing phosphate flux and the RE shuttle yield variable fluxes. However, even using the lowest possible estimates, a >100-fold difference in transport events is predicted between the C_4_ and C_3_ plant interfaces. The interface itself is probably optimized for a balance of open-ness to enable the flux and closed-ness to enrich the CO_2_ at the site of Rubisco, since different light intensities correlate with photosynthetic rates and plasmodesmatal density in *M. maximus* (Sowinski *et al.*, 2007). The fold change in transport events across the interface is in the range of the fold change expression changes for the C_4_ genes (Tables 2, 23). The evolution and hence also re-engineering of the C_4_ cycle must adapt the intercellular interface.

#### Accessory pathways to the core cycle

It is tempting to limit engineering efforts to the major transcriptional changes and therefore to the core cycle. However, accessory pathways to the core C_4_ cycle may play a major role in adapting the underlying metabolism to the presence of the carbon-concentrating pump.

The comparison of multiple different C_3_–C_4_ pairs and therefore C_4_ origins with each other provides a method to identify differentially regulated functions with biological significance, once the problem of paralogous genes carrying out the functions is overcome. By mapping RNA-Seq data to EC numbers and Pfam domains rather than individual genes, it has been possible to identify core C_4_ genes (Fig. 2; Supplementary Table S2 at *JXB* online), which indicates that these methods are suitable to pick up additional C_4_-related functions.

Both methods picked up functions involved in starch metabolism and sucrose synthesis (Fig. 2A, C). In the EC-based mapping, the sucrose synthesis pathway was present with two functions, the UDP-glucose pyrophosphorylase and the sucrose-phosphate synthase. Sucrose-phosphate synthase is the rate-limiting enzyme for sucrose synthesis in the C_3_ plant *A. thaliana* (Häusler *et al.*, 2000; Strand *et al.*, 2000; Koch, 2004) and NDP sugar pyrophosphorylases are comparatively slow enzymes. The surplus of fixed carbon (Fig. 1C, D) leads to a surplus of triose-phosphates. In *Z. mays*, *Panicum miliaceum*, and *Brachiaria erucaeformis*, sucrose synthesis is localized to the mesophyll (Usuda and Edwards, 1980), which may also be the case in *M. maximus*. Both the localization of sucrose synthesis and the higher carbon assimilation rate contribute to more triose-phosphate at the site of sucrose synthesis and hence the need for greater sequestration (Fig. 3). Similarly, the higher rate of CO_2_ assimilation (Fig. 2) and the localization of starch storage in the BSCs (Majeran and van Wijk, 2009; Majeran *et al.*, 2010) probably also require higher rates of starch synthesis to sequester the triose-phosphates efficiently (Figs 2, 3). When considering the engineering of C_4_ photosynthesis, the sequestration of triose-phosphates is probably of low priority compared with the engineering of the enzymes and transport proteins, yet not adding these functions for triose-phosphate sequestration will probably limit the system to the capacity of C_3_ photosynthetic plants, a 20% loss of potential productivity.

Insulating the C_4_ cycle from other metabolic networks is also probably critical to avoid loss of cycle intermediates. No obvious proteins with functions in this context were identified in comparisons across all C_4_ data sets (Fig. 2; Supplementary Table S2 at *JXB* online), although the uncharacterized functions may include such insulators (Supplementary Table S2). The analysis of only NAD-ME-based C_4_ photosynthesis registered changes, which represent the overlap between the dicot *C. gynandra* and the grass *M. maximus*. Both species produce pyruvate in their mitochondria (Table 2; Bräutigam *et al.*, 2011) and use aspartate as a dominant transfer acid. Both NAD-ME species show higher PDH kinase and reduced aspartate kinase and aspartate oxidase transcript amounts (Fig. 2). These three enzymes control metabolite exit from the C_4_ cycle as PDH kinase gates pyruvate decarboxylation for entry into the tricarboxylic acid (TCA) cycle, aspartate kinase controls entry into aspartate-derived amino acid metabolism, and aspartate oxidase controls entry into NAD synthesis. The leaking of cycle intermediates into other metabolism despite the insulation can be indirectly seen in the labelling pattern obtained by^ 14^CO_2_ feeding. If metabolites from the cycle are consumed, they need to be replaced from the CBB cycle and will thus carry label in C_2_–C_4_ of the four-carbon compounds and lead to label in the three-carbon compounds, which—if only cycling—should show no label at all. Indeed, labelling studies identified delayed labelling in both groups (e.g. Hatch, 1979), indicating that leaking of intermediates does occur. When engineering a C_4_ cycle into a C_3_ plant, limiting the leakage of cycle intermediates is probably required for all cycle metabolites to keep the cycle running robustly.

Two to three pathways are commonly down-regulated: the CBB cycle, photorespiration, and protein synthesis (Fig. 2). Reduced expression of these functions in C_4_ species may not be required to engineer efficient CO_2_ capture. However, reduced expression of CBB, photorespiration, and protein translation (Fig. 2) may be necessary to realize the nitrogen-saving benefits of C_4_ photosynthesis which are common to C_4_ plants (Sage, 2004).

NAD-ME species show an unusual pattern with regard to nucleotide metabolism; several functions of purine metabolism are up-regulated while several functions of pyrimidine synthesis are down-regulated. While one may speculate that the changes in purine metabolism are due to the altered ATP usage in these plants, the functional reason for these changes remains unknown.

Previous global transcriptome analyses found that genes encoding components of photosynthetic cyclic electron flow (CEF) were significantly up-regulated (Bräutigam *et al.*, 2011; Gowik *et al.*, 2011), raising the question of whether such alterations to photosynthesis are required in all C_4_ subtypes. The present analysis did not indicate differences in CEF in *M. maximus* compared with *D. clandestinum* (Supplementary Table S5 at *JXB* online). The reason lies in the high PEP-CK activity, which is fuelled by malate oxidation in mitochondria (Fig. 3). Malate is generated using photosynthetic REs leading to a 4:2.5 ATP:NADPH production ratio in photosynthesis which is very similar to that of C_3_ photosynthesis at a 3:2 ratio and in contrast to the classical C_4_ calculation of 5:2 (Kanai and Edwards, 1999). If considering engineering, a C_4_ cycle with high PEP-CK activity together with malate decarboxylation in mitochondria removes the requirement for dimorphic chloroplasts, which results in one less feature to be engineered.

It is tempting to think that the type of C_4_ photosynthesis realized in *M. maximus* is less efficient because of higher energy input for the C_4_ cycle (Fig. 3) and because of oxygen production in the bundle sheath, which increases the potential for photorespiration. Elevated photorespiration is indeed a feature of *M. maximus* (Furbank and Badger, 1982; Ohnishi and Kanai, 1983; Farineau *et al.*, 1984). However, the quantum efficiency of *M. maximus* is indistinguishable from that of *Z. mays* or *S. bicolor* (Ehleringer and Pearcy, 1983). It is surprising that the energy requirements derived from the model (Fig. 3) and the photorespiratory rate (Furbank and Badger, 1982; Ohnishi and Kanai, 1983; Farineau *et al.*, 1984) do not predict quantum efficiency.

The blueprint of C_4_ metabolism in *M. maximus* is simpler compared with that of NAD-ME and NADP-ME plants, because the generation of transfer acids requires fewer adjustments in intracellular transport capacity and photosynthetic electron transfer, and at least some part of the insulators that prevent leakage of C_4_ cycle intermediates into general metabolism are known. Thus, it represents an attractive target for engineering the C_4_ cycle into a C_3_ crop plant.

## Supplementara data

Supplementary data are available at *JXB* online.

Figure S1. Cross-sections of *D. clandestinum* and *M. maximus.*

Table S1. *D. clandestinum* and *M. maximus* unigene fasta files.

Table S2. Excel table of Pfam and EC function analysis for all genes.

Table S3. Excel table of quantitative gene expression information including statistical analysis.

Table S4. Text document of selected full-length unigenes including alignment to *S. italica* genes and targeting prediction.

Table S5. Excel table of enrichment analysis for pathways.

## Supplementary Material

Supplementary Data
