# Supplementary material for: Towards an integrative model of C4 photosynthetic subtypes: insights from comparative transcriptome analysis of NAD-ME, NADP-ME, and PEP-CK C4 species
Source: J Exp Bot. 2014 Mar 18;65(13):3579–93. doi: 10.1093/jxb/eru100 (PMC4085959; doi:10.1093/jxb/eru100)
Supplement: Supplementary Data [file supp_65_13_3579__index.html]

Towards an integrative model of C4 photosynthetic subtypes: insights from comparative transcriptome analysis of NAD-ME, NADP-ME, and PEP-CK C4 species — Supplementary Data 

# Towards an integrative model of C4 photosynthetic subtypes: insights from comparative transcriptome analysis of NAD-ME, NADP-ME, and PEP-CK C4 species

## Supplementary Data

Data files

**Files in this Data Supplement:**

- Supplementary Data - Supplementary Data
- Supplementary Data - Supplementary Data
- Supplementary Data - Supplementary Data
- Supplementary Data - Supplementary Data
- Supplementary Data - Supplementary Data
