# Supplementary material for: Towards an integrative model of C4 photosynthetic subtypes: insights from comparative transcriptome analysis of NAD-ME, NADP-ME, and PEP-CK C4 species
Source: J Exp Bot. 2014 Mar 18;65(13):3579–93. doi: 10.1093/jxb/eru100 (PMC4085959; doi:10.1093/jxb/eru100)
Supplement: Supplementary Data [file supp_eru100_jexbot116442_file005.pdf]

Towards an integrative model of C_4 photosynthetic subtypes – insights from comparative transcriptome analysis of NAD-ME, NADP-ME, and PEP-CK C_4 species . Andrea Bräutigam, Simon Schliesky, Canan Külahoglu, Colin Osborne, and Andreas P M Weber. *Journal of Experimental Botany 2014*

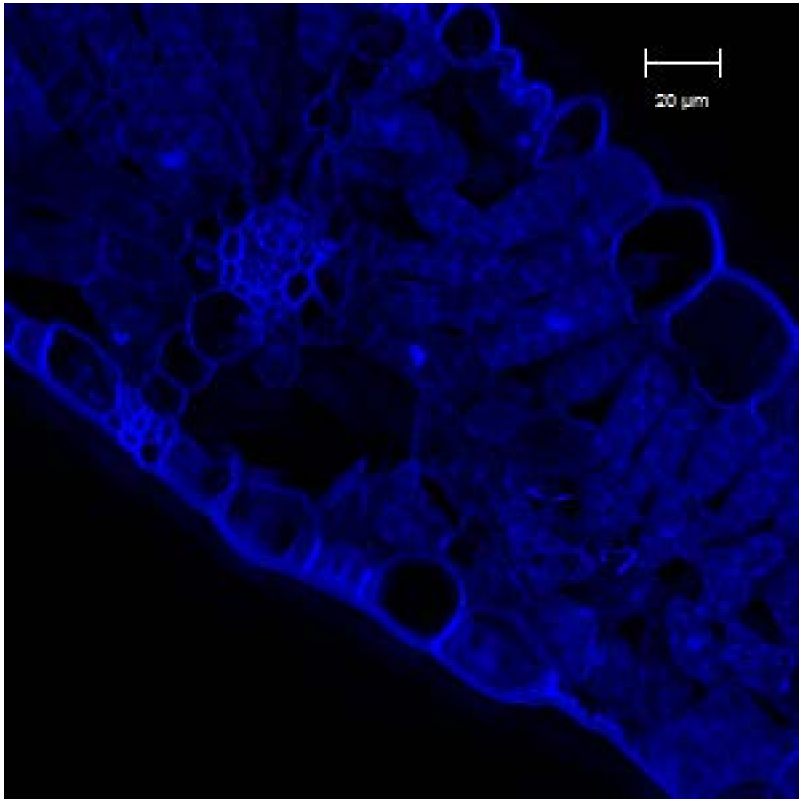

Dichanthelium clandestinum (C_3)

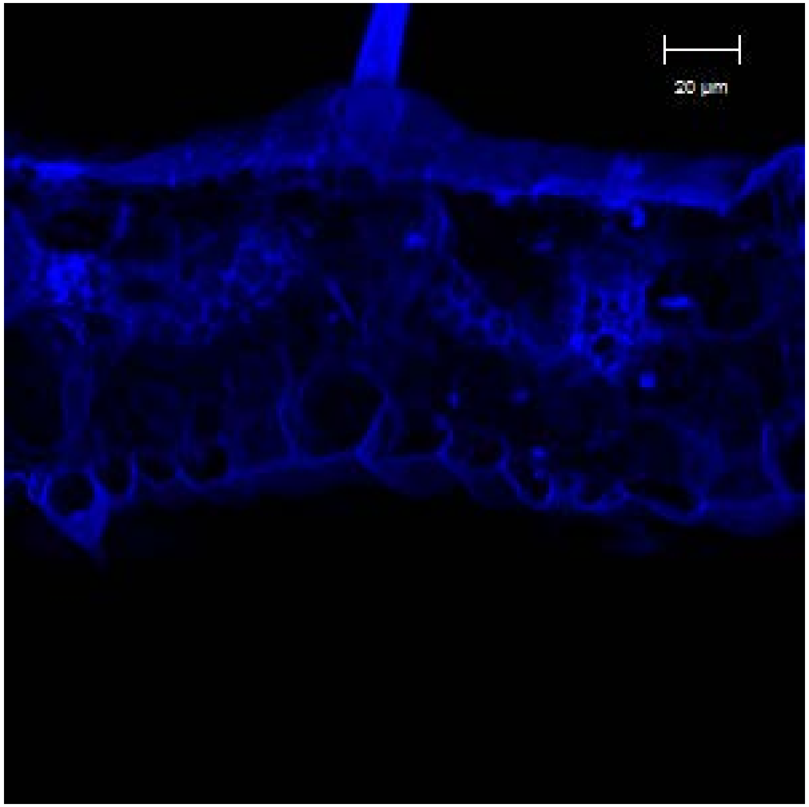

Megathyrsus maximus (C_4)
